# Silver Ions Inhibit Bacterial Movement and Stall Flagellar Motor

**DOI:** 10.3390/ijms241411704

**Published:** 2023-07-20

**Authors:** Benjamin Russell, Ariel Rogers, Ryan Yoder, Matthew Kurilich, Venkata Rao Krishnamurthi, Jingyi Chen, Yong Wang

**Affiliations:** 1 Department of Physics, University of Arkansas, Fayetteville, AR 72701, USA; 2 Department of Chemistry and Biochemistry, University of Arkansas, Fayetteville, AR 72701, USA; 3 Materials Science and Engineering Program, University of Arkansas, Fayetteville, AR 72701, USA; 4 Cell and Molecular Biology Program, University of Arkansas, Fayetteville, AR 72701, USA

**Keywords:** hidden Markov model, antibiotics, *E. coli*, motility, tethering assay, rotation

## Abstract

Silver (Ag) in different forms has been gaining broad attention due to its antimicrobial activities and the increasing resistance of bacteria to commonly prescribed antibiotics. However, various aspects of the antimicrobial mechanism of Ag have not been understood, including how Ag affects bacterial motility, a factor intimately related to bacterial virulence. Here, we report our study on how Ag^+^ ions affect the motility of *E. coli* bacteria using swimming, tethering, and rotation assays. We observed that the bacteria slowed down dramatically by >70% when subjected to Ag^+^ ions, providing direct evidence that Ag^+^ ions inhibit the motility of bacteria. In addition, through tethering and rotation assays, we monitored the rotation of flagellar motors and observed that the tumbling/pausing frequency of bacteria increased significantly by 77% in the presence of Ag^+^ ions. Furthermore, we analyzed the results from the tethering assay using the hidden Markov model (HMM) and found that Ag^+^ ions decreased bacterial tumbling/pausing-to-running transition rate significantly by 75%. The results suggest that the rotation of bacterial flagellar motors was stalled by Ag^+^ ions. This work provided a new quantitative understanding of the mechanism of Ag-based antimicrobial agents in bacterial motility.

## 1. Introduction

The rising prevalence of antibiotic resistance in harmful microbes due to the overuse of conventional antibiotics has become a serious global concern for public health [1,2,3], posing the need for different approaches for fighting against drug-resistant microbes [4,5]. Recent research in the past two decades has revisited the antimicrobial activities of noble metals, such as silver (Ag), in different forms—including ions and nanoparticles—and has uncovered their strong capacity for suppressing bacterial growth and killing bacteria [6,7,8]. Exciting progress has been made towards understanding the antimicrobial mechanism of Ag, suggesting that Ag causes multimodal damages to bacteria, including DNA damage, membrane disruption, free radical generation (ROS), and loss of ATP production [7,9,10,11,12,13]. However, various aspects of the antimicrobial mechanism of Ag remain elusive, and the temporal resolution for understanding Ag-caused damages in bacteria is still limited [7,14,15], including how Ag affects the motility of bacteria, which is tightly coupled with bacterial virulence [16].

Motility is essential to many bacteria for detecting and pursuing nutrients, as well as avoiding and fleeing from toxicants. Certain bacteria, such as *Escherichia coli* (*E. coli*), use flagella to move in aqueous environments [17]. *E. coli* flagella are filaments extending outward from the bacteria [18]. The flagella are connected to and driven by motors embedded in the bacterial membrane through hooks [19]. For *E. coli* and other peritrichous bacteria with flagella covering their entire surfaces, their movement depends on the rotation direction of their flagella [17,19]. When flagella rotate counterclockwise (CCW), they are bundled and propel the bacteria to move directionally (i.e., running) for purposeful movement toward chemical attractants or away from repellents [17,20]; when flagella rotate clockwise (CW), they are splayed out, resulting in reorientation (i.e., tumbling) of the bacteria [17,20]. In addition, flagellar rotation may stop intermittently (i.e., pausing) [21,22]. The *E. coli* flagella contain mainly three parts: the filament; the hook; and the basal body [17,20]. The basal body consists of several rings, some of which (e.g., MS ring and C ring) are essential components of the flagellar motor for driving the rotation of the flagella [17]. Structurally, the flagellar motor involves both the stator proteins (e.g., MotA and MotB) and the rotor proteins (e.g., FliG, FliM, and FliN), which also play critical roles in the torque generation of the motor [17]. Functionally, the CW/CCW direction of the flagellar motor’s rotations relies on another set of chemotaxis proteins (e.g., CheY, CheZ, CheA, CheW, CheR, and CheB). For example, the flagellar motor switches from CCW rotation to CW rotation when the phosphorylated response regulator CheY binds to the flagellar motor [23].

As Ag in various forms (e.g., ions, nanoparticles) suppresses and kills bacteria, we hypothesized that the motility of bacteria is significantly affected by Ag. This hypothesis is indirectly supported by evidence from previous studies. For example, Ivask et al. performed liquid-culture-based high-throughput growth assays for a library of single-gene-deletion strains of *E. coli* and found that a series of flagella-related mutants (e.g., *fliG*, *fliM*, *flgF*, *flgG*, etc., which are involved in the assembly and function of flagella) were sensitive to Ag^+^ ions and Ag nanoparticles [13]. Also, plate-based chemical-genetic-screening assays on a similar library identified and confirmed some flagella-related genes (e.g., *flgA*, *flgD*, *flgJ*, *flgK*, *fliC*, *fliE*, *fliL*, *fliP*, *fliR*, and *motB*) [24]. In addition, recent work by us and others showed that Ag affects the organization and function of certain universal regulatory proteins in bacteria, such as histone-like nucleoid structuring (H-NS) proteins, which regulate bacterial chemotaxis and motility [14,24,25,26,27]. Furthermore, plate-based swimming and swarming motility assays suggested that Ag could change the motility of bacteria under certain conditions [28]. Lastly, Ag^+^ ions have been used to stain bacterial flagella for decades [29], implying that Ag^+^ ions interact with flagella.

However, few studies on real-time observation and quantification of Ag’s effects on bacterial movement are reported in the literature [7,30]. In this work, we investigated the effects of Ag^+^ ions on the swimming behavior of *E. coli* bacteria based on microscopic imaging, with a temporal resolution of 15–50 ms. Ag^+^ ions were chosen for two reasons. First, Ag^+^ ions are effective at suppressing and killing bacteria [6,10,31]. Second, the release of Ag^+^ ions from Ag nanoparticles is a major contribution to the toxicity of Ag nanoparticles [7]. Through swimming assays, we provided direct evidence that Ag^+^ ions inhibit the motility of bacteria. In addition, we monitored the rotation of flagellar motors of *E. coli* bacteria using tethering and rotation assays in the absence and presence of Ag^+^ ions, directly observing that Ag^+^ ions increased the frequency of bacterial tumbling/pausing. Furthermore, based on hidden Markov model (HMM) analysis, we found that Ag^+^ treatment caused the bacterial transition rate from the tumbling/pausing state to the running state to decrease significantly, suggesting that the rotation of bacterial flagellar motors was stalled by Ag^+^ ions. These quantifications and analysis based on high-temporal-resolution microscopic imaging provide direct evidence of Ag’s effects on bacterial mobility.

## 2. Results and Discussions

### 2.1. Lower Motility of Bacteria Caused by Ag^+^ Ions

**Figure 1:**
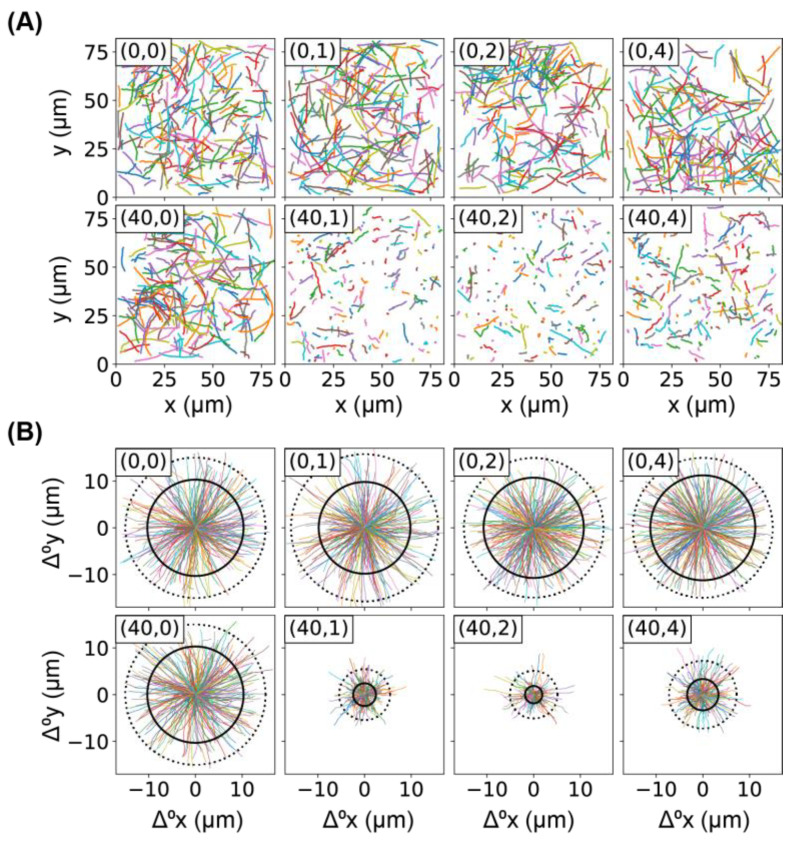
Motion of bacteria in the absence and presence of Ag^+^ ions. (**A**) Trajectories of bacteria, untreated or treated by Ag^+^ ions at 40 µM. Each sub-figure contains 200 randomly chosen trajectories and is labeled by (c_Ag_, T_tr_), where c_Ag_ is the concentration of Ag^+^ ions and T_tr_ is the treatment/incubation time. (**B**) Rose graphs of the first 12 frames of trajectories of bacteria, untreated or treated by Ag^+^ ions at 40 µM. Each sub-figure is labeled similarly as in (**panel A**). Under each condition, 300 randomly chosen examples of the trajectories were shown in color, while the mean and 90th percentile of the displacements of the first 12 frames of all the trajectories were shown as solid and dotted circles, respectively.

We first examined the effects of Ag^+^ ions on the motility of *E. coli* bacteria using swimming assays [32,33,34] as described in the “Materials and Methods” section (Section 3). We observed that, compared to the untreated bacteria (i.e., 0 h), bacteria treated with Ag^+^ ions at 40 µM for 1, 2, and 4 h were much slower (Movies S1 and S2). From the movies of the freely swimming bacteria, the trajectories r(t) of individual bacteria were obtained. A total of 200 randomly chosen examples of trajectories for each experimental condition are shown in Figure 1A, where longer traveling distances were observed for the untreated bacteria compared to the ones treated with Ag^+^ ions. To see this difference more clearly, we plotted the corresponding rose graphs [35], in which the displacements of the bacteria from their individual initial positions were drawn, $\Delta r^{0}\left(t\right)=r\left(t\right)-r\left(0\right)$. A total of 300 randomly chosen examples were shown in Figure 1B, where the first 12 frames of the trajectories were shown to eliminate the differences due to different lengths of trajectories [35]. Note that a length of 12 was the minimum number of frames of trajectories to be included in the analysis, while many trajectories were much longer (~70 frames). It is obvious that the motility of bacteria decreased significantly after the treatment with Ag^+^ ions. We quantified the mean and 90th percentile of the displacements of the first 12 frames of all the trajectories in each condition, shown as solid and dotted circles in the rose graphs (Figure 1B), respectively. We found that the two circles for untreated bacteria did not change significantly from 0 to 4 h, indicating that the motility of the bacteria remained similar. In contrast, the treated bacteria showed much smaller radii for both the mean ($\bar{\Delta^{0}r}$) and 90th percentile circles, indicating that Ag^+^ treatment led to lower bacterial motility. We also noted that the radii slightly increased with a longer treatment time, implying possible recovery of the bacteria as reported by previous results [6,15].

**Figure 2:**
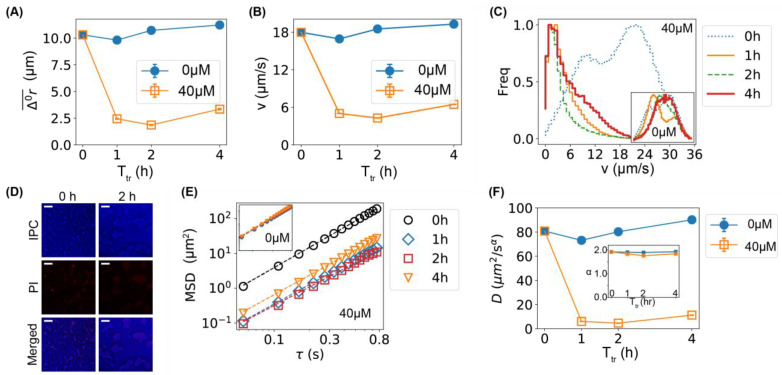
Lower motility of bacteria caused by Ag^+^ ions. (**A**) The dependence of the mean displacements ($\bar{\Delta^{0}r}$) of the first 12 frames of all trajectories of bacteria on incubation/treatment time in the absence (0 µM) and presence of Ag^+^ ions (40 µM). (**B**) The dependence of the mean bacterial velocity on incubation/treatment time in the absence (0 µM) and presence of Ag^+^ ions (40 µM). (**C**) Distributions of bacterial velocities in the presence of Ag^+^ ions at 40 µM for 0, 1, 2, and 4 h. Inset: the corresponding result for untreated bacteria (0 µM). The axes of the inset are the same as the main figure. (**D**) Cell-viability assay based on propidium iodide (PI) staining for untreated (0 h, left column) and treated (2 h, right column) bacteria. Top: inverted phase-contrast (IPC) images; middle: fluorescence images due to PI staining; bottom: merged IPC/PI images. Scale bar = 16 µm. (**E**) Log–log plot of mean-square displacements (MSDs) vs. lag time (τ) for trajectories of treated bacteria by Ag^+^ ions at 40 µM for 0, 1, 2, and 4 h. Inset: the corresponding result for untreated bacteria (0 µM). (**F**) Dependencies of the generalized diffusion coefficient D and the anomalous scaling exponent α (inset) on the incubation/treatment time T_tr_.

The slower motion of bacteria caused by Ag^+^ ions was further visualized in Figure 2A by plotting the radii of the mean circles in the rose graphs ($\bar{\Delta^{0}r}$, Figure 1B) as functions of the treatment time. To further confirm that the Ag^+^ ions inhibited the movement of bacteria, we calculated the instantaneous velocities of the bacteria directly from the trajectories, v=|v|=|Δr/Δt|, where Δt= 0.054 s is the time interval between adjacent frames. The dependence of the mean velocity on the treatment time is shown in Figure 2B, showing the same trends as $\bar{\Delta^{0}r}$. The average velocity decreased by >70% after the bacteria were treated by Ag^+^ ions for 1 or 2 h. In addition, we examined the distributions of the bacterial velocities (Figure 2C) and observed a double-peak distribution (centered around 10 and 22 µm/s) for the untreated bacteria (*t* = 0 h), while Ag^+^ treatment moved the peak to ~2 µm/s (Figure 2C). Such a significant shift in the velocity distribution was absent in the negative controls (inset of Figure 2C).

As the bacterial velocities were reduced to 0 (peaked at ∼2 µm/s) after Ag^+^ treatment, one possibility is that the bacteria were killed by Ag^+^ ions at 40 µM. However, the chance of this possibility was low for the following reasons. First, our previous work showed that the majority of bacteria treated with 60 µM Ag^+^ ions were alive, fighting against damages caused by Ag^+^ ions and showing oscillations in their cell lengths within 12 h [15]. Second, the cell-viability assay based on propidium iodide staining [36] showed that the number of dead cells (red cells) was low and the majority of treated bacteria were alive at 40 µM Ag^+^ ions (Figure 2D). Third, if the bacteria were killed, they would display random diffusion (Brownian motion), and the corresponding mean-square displacement (MSD) would be proportional to the diffusion coefficient (D) and the lag time (τ), giving a slope of 1 in the log–log plot of MSD vs. τ (Figure 2E) [37,38]. However, fitting the experimental MSD curves (Figure 2E) with $MSD=4D\tau^{\alpha }$ (α is the anomalous scaling exponent) showed that α remained ≈2 in the presence of Ag^+^ ions for various durations of treatment time (inset of Figure 2F), indicating that the bacteria retained active motion after Ag^+^ treatment [39,40]. In contrast, the fitted constant D decreased significantly (Figure 2F), following the same dependence on treatment time as the mean velocity of the bacteria (Figure 2A,B). It should be noted that the fitted D values were not the diffusion coefficients as the exponent α is close to 2; the purpose of the MSD analysis was to demonstrate that the Ag^+^-caused slower motion of the bacteria was different from diffusion.

### 2.2. Comparison of Bacterial Movement before and after Ag^+^ Treatment

**Figure 3:**
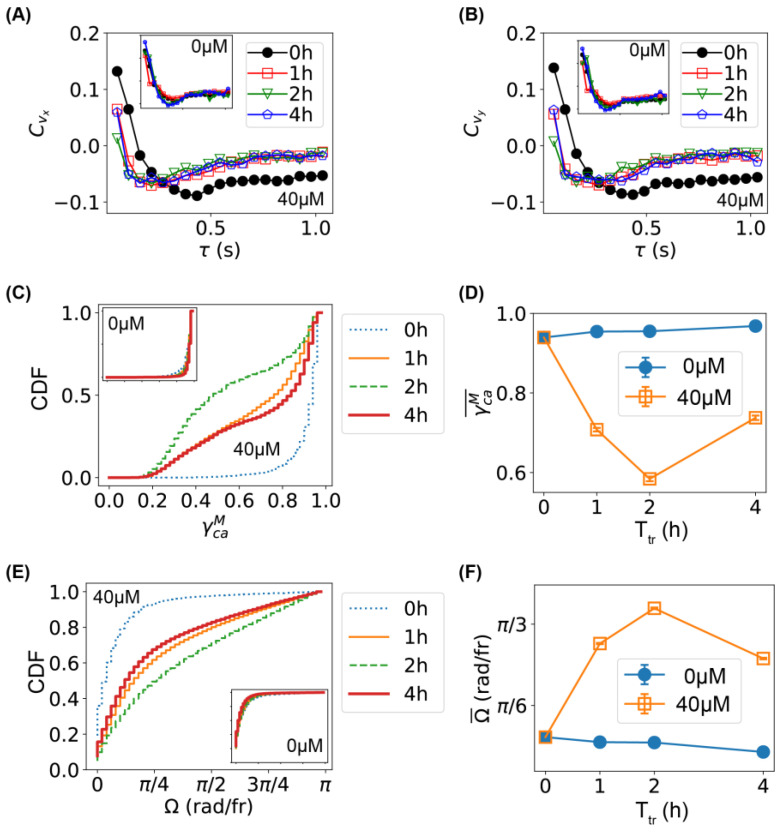
Characterization of bacterial movement and comparison between untreated and treated bacteria. (**A**,**B**) Autocorrelation of velocities (**A**: v_x_; **B**: v_y_) for bacteria treated with Ag^+^ ions at 40 µM for 0, 1, 2, and 4 h. Insets: the corresponding results for untreated bacteria. (**C**) Cumulative distribution function (**C**,**D**,**F**) of the maximum chord-to-arc ratio ($\gamma_{CA}^{M}$) for the trajectories of bacteria untreated (0 h) or treated with 40 μM Ag^+^ ions for 1, 2, and 4 h. (**D**) Dependence of the mean of $\gamma_{CA}^{M}$ on treatment time. (**E**) CDF of the changing rate of swimming directions (Ω) for bacteria untreated (0 h) or treated with 40 μM Ag^+^ ions for 1, 2, and 4 h. (**F**) Dependence of the mean of Ω on treatment time.

We quantitatively compared the movement of bacteria before and after Ag^+^ treatment by examining the velocity autocorrelation. Briefly, we calculated the autocorrelations of the x and y components of bacterial velocities, $C_{v_{i}}\left(\tau \right)=\frac{\langle v_{i}\left(t+\tau \right)v_{i}\left(t\right)\rangle }{\langle v_{i}\left(t\right)v_{i}\left(t\right)\rangle }$, where $v_{i}=v_{x}$ or $v_{y}$ and τ is the lag time. For untreated bacteria, velocity autocorrelation did not change at different incubation times (insets of Figure 3A,B); in contrast, treating the bacteria with Ag^+^ ions resulted in shifts to the left in the velocity autocorrelation (Figure 3A,B). The left shift of the velocity autocorrelation suggests that the “persistence” time of the bacterial movement became shorter after Ag^+^ treatment and the movement of bacteria became less straight than that before treatment.

We also examined the maximum chord-to-arc ratio ($\gamma_{CA}^{M}$) of the trajectories (inspired by TumbleScore [35]): $\gamma_{CA}^{M}=C^{M}/A$, where $C^{M}=max_{i,j}\left(\left|r_{i}-r_{j}\right|\right)$ is the maximum chord length of a trajectory and $A=\sum_{i}\left|r_{i+1}-r_{i}\right|$ is the “arc” length of the trajectory. If a trajectory is straight, $\gamma_{CA}^{M}\approx 1$, while a trajectory dominated by directional changes gives $\gamma_{CA}^{M}\approx 0$; therefore, the maximum chord-to-arc ratio could be used as another indicator of the persistence of the trajectories. The cumulative distributions (CDFs) of the $\gamma_{CA}^{M}$ of all the trajectories for bacteria untreated (0 µM and/or 0 h) or treated with Ag^+^ ions for 1, 2, and 4 h are shown in Figure 3C. Compared to the untreated bacteria, the CDFs for treated bacteria rose up at lower $\gamma_{CA}^{M}$ values, indicating that Ag^+^ ions led to higher fractions of lower $\gamma_{CA}^{M}$. This change was obvious by examining the time dependence of the mean values of $\gamma_{CA}^{M}$ (Figure 3D). We note that a similar result was observed for the normalized maximum chord-to-arc ratio $\beta_{CA}^{M}=\gamma_{CA}^{M}/N$ where N is the length of the trajectory.

Furthermore, we estimated the changing rate of moving directions directly from the trajectories, $\Omega =cos^{-1}\left(v_{i+1}\cdot v_{i}/v_{i+1}v_{i}\right)$ [35,41,42]. The CDFs of Ω for all the trajectories of bacteria untreated (0 µM and/or 0 h) or treated with Ag^+^ ions for 1, 2, and 4 h are shown in Figure 3E. We found that the CDFs lowered down after Ag^+^ treatment, indicating an increased fraction of higher Ω values. This was confirmed by the time dependence of the mean values of Ω (Figure 3F). All three quantifications ($C_{v}$, $\gamma_{CA}^{M}$, and Ω) showed consistent results that the movement of bacteria became less persistent (i.e., less straight) after subjecting the bacteria to Ag^+^ ions.

### 2.3. Higher Frequency of Tumbling/Pausing Caused by Ag^+^ Ions in Tethering Assays

**Figure 4:**
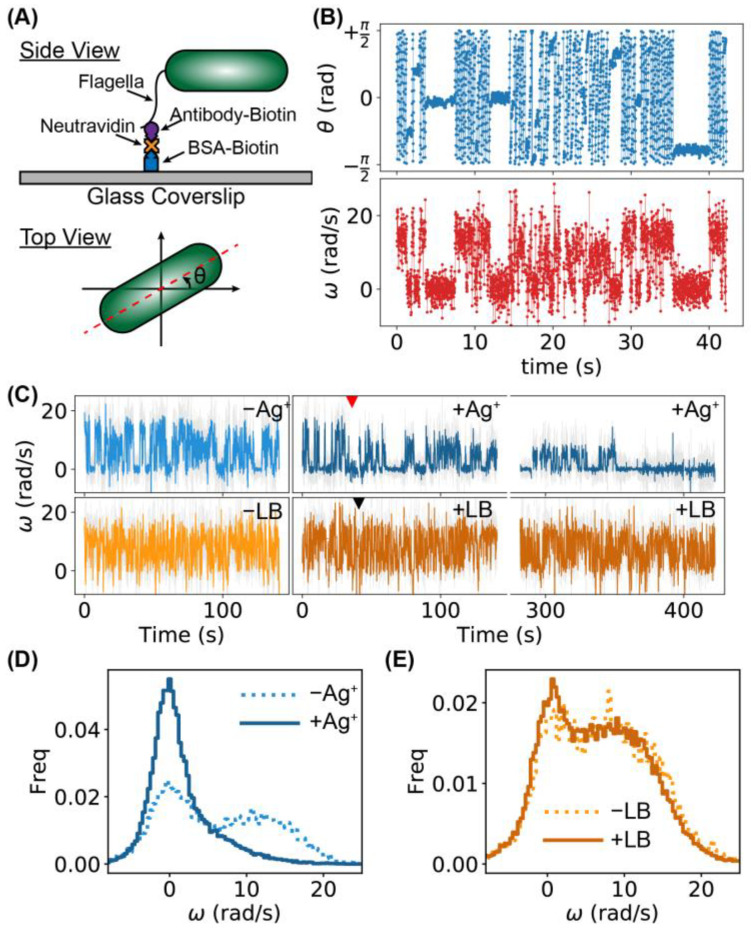
Tethering assay for investigating the running and tumbling/pausing of individual bacteria. (**A**) Tethering of a bacterium on a glass coverslip (side view) and orientation of a bacterium θ (top view). (**B**) Examples of trajectories of orientation θ and angular velocity ω of a bacterium for 3000 frames (or 42.3 s). (**C**) Examples of ω-trajectories for two bacteria. The top one was treated (blue curves) with Ag^+^ ions; the red arrow indicates the time of adding Ag^+^ ions. The bottom trajectories (orange curves) were for a bacterium without treatment. LB medium was added into the sample at the time indicated by the black arrow. (**D**) Distributions of ω for a bacterium treated by Ag^+^ ions: pre-Ag^+^ (dotted) and post-Ag^+^ (solid). (**E**) Distributions of ω for an untreated bacterium: pre-LB (dotted) and post-LB (solid).

To further understand the underlying mechanism of the inhibition of bacterial motility by Ag^+^ ions, we performed the tethering assay on individual bacteria [43,44], which has been used extensively in the literature [21,22], and was achieved using biotinylated Anti-FliC antibody, neutravidin, and biotinylated bovine serum albumin (BSA) in the current study (Figure 4A) [45]. The tethering assay was chosen in this study for several reasons, while other untethered rotation assays (e.g., using shearing or mutation) are available [45,46,47,48,49]. First, the same individual bacterium could be compared directly before and after adding Ag^+^ ions, as many of the tethered bacteria did not move away after the addition of Ag^+^ ions. Second, the bacteria remained intact, while significant portions of the flagellar filaments were removed in sheared bacteria [45,46,47,48,49]. Third, the same strain of bacteria was used in both the tethering assay and the swimming assay, yielding a direct comparison with the swimming assay. On the other hand, we noted that the current method is likely to introduce multiple tethering points for a single bacterium, which may make the pausing state more noticeable than untethered bacteria [21,22,47,48,49,50].

The tethered bacteria rotated on the glass surfaces as the flagellar motors rotated (Movie S3) [45,51]. Between continuous rotations (i.e., running), occasional pauses and reversed rotations were observed, corresponding to the pausing and tumbling of the bacteria (Movie S3) [46,52]. After adding Ag^+^ ions to the bacteria, it was observed that the rotation of the bacteria slowed down, and the frequency of pausing increased (Movie S4).

To quantify the results of the tethering assay, we first extracted the orientation of the bacteria, θ∈(−π/2,+π/2] in each frame of the movies, then the angular velocities of the bacterial rotations were calculated as ω=Δθ/Δt, where Δθ and Δt=0.0141 s are the change in the bacterial orientation and time interval between adjacent frames, respectively. Examples of trajectories of θ and ω for 3000 frames (or 42.3 s) for a bacterium before Ag^+^ treatment are shown in Figure 4B. Two distinct states were observed in the ω-trajectory, presumably corresponding to the running and tumbling/pausing states [46,52]. The full ω-trajectory (10,000 frames) of Figure 4B is shown in Figure 4C (-Ag^+^, light blue), while two segments (each with 10,000 frames) of the ω-trajectory of the same bacterium during and after Ag^+^ treatment are also presented (Figure 4C, +Ag^+^, dark blue), where the red arrow indicates the time of adding Ag^+^ ions. The tumbling/pausing state (i.e., lower angular velocity) became more frequent after Ag^+^ treatment. In contrast, untreated bacteria (adding LB medium instead of Ag^+^ ions) did not show observable differences in the ω-trajectories (Figure 4C, ±LB, light and dark orange). This observation was quantified by the distribution of the angular velocities. For the control, double peaks were observed both before and after the addition of LB medium (Figure 4E); in contrast, the tumbling/pausing peak (lower ω) became dominant after the addition of Ag^+^ ions (Figure 4D). The observed increase in the tumbling/pausing frequency is consistent with a previous report based on swimming assays for the effect of Ag nanoparticles [30].

### 2.4. Higher Frequency of Tumbling/Pausing Caused by Ag^+^ Ions in Untethered Rotation Assays

Although the tethering assay was preferred in this study due to its advantage of allowing us to observe the same bacteria before and after the addition of Ag^+^ ions and to make direct comparisons, one concern is that multiple flagella filaments may be tethered to the glass surfaces. Tethering by multiple points may make the pausing state more noticeable than untethered bacteria. To address this concern, we performed untethered rotation assays (Movie S5) using bacteria with shortened flagella by shearing [47,49,50,53], which showed similar results to the tethering assay.

**Figure 5:**
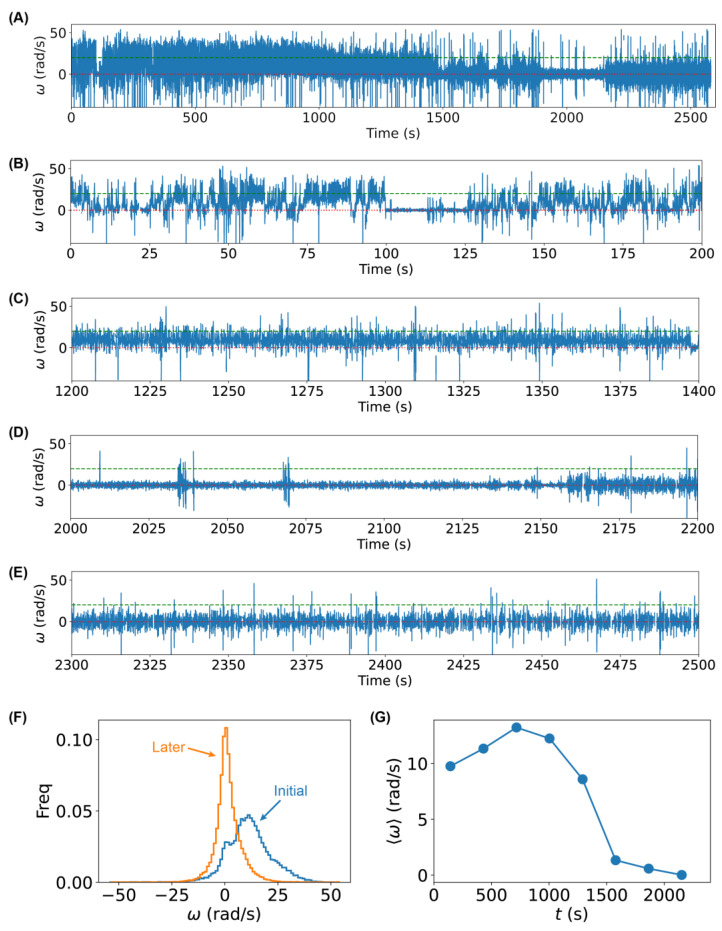
Higher frequency of tumbling/pausing caused by Ag^+^ ions using untethered rotation assay. (**A**) Angular velocity ω trajectory of 2600 s of a single bacterium with flagellar filaments shortened by shearing after adding Ag^+^ ions to the solution at time = 0 s. (**B**–**E**) Zoom-in of the angular velocity trajectory in different ranges of treatment time. Red dotted lines and green dashed lines highlight the values of 0 and 20 rad/s, respectively. (**F**) Distributions of the angular velocity ω for the same bacterium for the initial ~1300 s (blue) or later ~1300 s (orange) after adding Ag^+^ ions. (**G**) Mean angular velocity 〈ω〉 (averaged over ~287 s) as a function of treatment time.

We observed that the magnitude of the angular velocity of the bacteria decreased after adding Ag^+^ ions (Figure 5). For example, the angular velocity of the bacterium in the running state was ~20 rad/s initially, while it decreased to ~10 rad/s after ~1000 s (Figure 5A–E). In addition, longer pauses were observed (Figure 5A–E), indicating that the flagellar motors indeed stopped intermittently without tethering. Furthermore, we examined the distribution of the angular velocity and found that the tumbling/pausing peak (lower ω) became dominant during the latter half of the trajectory (Figure 5F). Such an observation was further confirmed by examining the running average of the angular velocity as a function of the treatment time (Figure 5G). These observations were consistent with those from the tethering assay, suggesting that the tethering assay could reliably measure the flagellar motor motion. On the other hand, we noted that, compared to the tethering assay, bacterial tumbling (i.e., rotation in the CW direction) was observed more frequently in the untethered rotation assay, implying that multiple flagellar filaments were attached in the antibody-based tethering assay.

### 2.5. Stalling of Flagellar Motors Caused by Ag^+^ Ions

**Figure 6:**
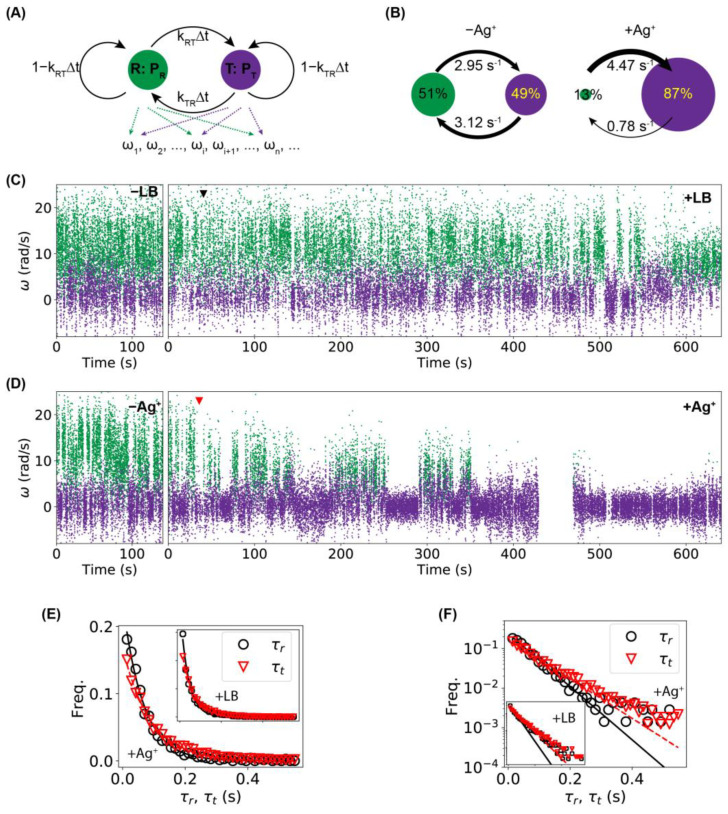
Hidden Markov model (HMM) analysis. (**A**) The hidden Markov model with two states (running (R) vs. tumbling/pausing (T)), which emit observations of angular velocities $\omega_{i}$. The probabilities for the system to be in the running (green) and tumbling/pausing (purple) states are $P_{R}$ and $P_{T}$, respectively. The transition probabilities between the two states are $P_{RT}=k_{RT}\Delta t$ and $P_{TR}=k_{TR}\Delta t$, where $k_{RT}$ and $k_{TR}$ are the corresponding transition rates and Δt is the time interval between observations. (**B**) Predicted parameters ($P_{R}$, $P_{T}$, $k_{RT}$, and $k_{TR}$) from the HMM analysis for pre-Ag^+^ and post-Ag^+^ ω-trajectories of the bacterium in the top row of Figure 4C. (**C**,**D**) Predictions of states from the fitted/trained HMM model for the angular velocity (ω) trajectories for (**C**) an untreated bacterium and (**D**) a Ag^+^-treated bacterium. Green and purple colors indicate the running and tumbling/pausing states, respectively. The red and black arrows indicate the time of adding Ag^+^ ions or LB medium, respectively. (**E**,**F**) Distributions of the dwell times ($\tau_{r}$ for running dwell time and $\tau_{t}$ for tumbling/pausing dwell time) from the untreated (insets) and Ag-treated bacteria shown in panels (**C**) and (**D**) in (**E**) linear scale or (**F**) log-linear scale. Solid and dashed lines are fitted exponential curves.

To obtain a deeper understanding of why Ag^+^ ions inhibit bacterial movement and induce higher tumbling/pausing frequency, we performed hidden Markov model (HMM) analysis [54] on the trajectories of angular velocities. Results from the tethering assays were used in this analysis due to its advantage of allowing direct comparison on the same bacteria before and after exposure to Ag^+^ ions. It was noted that the hidden Markov model is necessary because the motility states of the bacteria were not directly measured from the experiments; instead, the observable value (i.e., the directly measured quantity) was the angular velocity (ω). Also, for simplicity, we only considered the running state vs. non-running state, without distinguishing the pausing state from the tumbling state. Therefore, our hidden Markov model assumes two states, namely a running state (R) and a tumbling/pausing state (T), which emit observations of angular velocities (Figure 6A). The probabilities for a bacterium to be in the running and tumbling/pausing states are $P_{R}$ and $P_{T}$, respectively. The bacterium can switch between the two states, with transition rates of $k_{RT}$ (from R to T) and $k_{TR}$ (from T to R). For a given time interval between observations (Δt=0.0141 s between adjacent frames in the tethering assay) that is much shorter than the typical switching dynamics (0.2–4 s) [47,48,50], the transition probabilities would be $P_{RT}=k_{RT}\Delta t$ and $P_{TR}=k_{TR}\Delta t$. For each bacterium, we fitted/trained the HMM using the pre-Ag^+^ or pre-LB data, and the fitted model was used to predict the states of all the observed angular velocities for that bacterium, which were then used to estimate the HMM parameters (P’s and k’s). As an example, the predicted states and the HMM parameters ($P_{R}$, $P_{T}$, $k_{RT}$, and $k_{TR}$) for the ±Ag^+^ bacterium in Figure 4C are presented in Figure 6C and Figure 6B, respectively. Two significant changes were observed. First, the tumbling/pausing probability ($P_{T}$) increased dramatically from 49% to 87% (correspondingly, $P_{R}=1-P_{T}$ decreased); second, while the running-to-tumbling/pausing transition rate increased slightly, the tumbling/pausing-to-running transition rate $k_{TR}$ decreased significantly after Ag^+^ treatment (3.12 $s^{-1}$ to 0.75 $s^{-1}$, a decrease of 75%). These observations suggest that Ag^+^ ions lead to higher tumbling/pausing frequency by blocking the transition from the tumbling/pausing state to the running state.

As simple hidden Markov models typically assume exponential distributions for dwell times (i.e., the time staying in the states), we wondered whether and how this assumption was satisfied in the tethering assay. Briefly, from the predicted states for the control and sample shown in Figure 6C (+LB and +Ag^+^, respectively), we calculated the running time ($\tau_{r}$) and tumbling/pausing time ($\tau_{t}$) and found that the distributions of both dwell times followed roughly the exponential distribution for both the control (+LB) and the sample (+Ag^+^), as shown in Figure 6D, where the solid and dashed lines are fittings. This observation indicates that the hidden Markov model is reasonably suitable for the analysis here. On the other hand, we noted that a closer look at the distributions of the dwell times in the log–linear scale indicated that a single exponential decay did not fit the data well (Figure 6E), suggesting that modified hidden Markov models that assume arbitrary distributions of dwell times may improve analysis.

**Figure 7:**
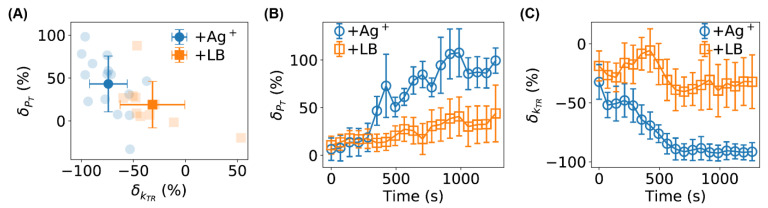
(**A**) Statistics of the relative changes in $P_{T}$ and $k_{TR}$ for 10 untreated (orange squares) and 15 Ag^+^-treated bacteria (blue circles). Error bars stand for standard deviation. (**B**,**C**) Time dependencies of the relative changes in (**B**) $P_{T}$ and (**C**) $k_{TR}$ for untreated (orange squares) and Ag^+^-treated (blue circles) bacteria. Error bars stand for the standard error of the mean.

We replicated the tethering assay experiments and HMM analysis on 10 untreated (±LB) and 15 treated (±Ag^+^) bacteria. We observed large variations in the absolute values of the angular velocities for different bacteria, which could be attributed to differences in cell length, the number of tethered flagella per bacterium, and the location of tethering points on the flagella [45,55]. To compare among different bacteria, we used the relative changes in the HMM parameters, $\delta_{P_{T}}=\left(P_{T}^{+}/P_{T}^{-}-1\right)\times 100\%$ and $\delta_{k_{TR}}=\left(k_{TR}^{+}/k_{TR}^{-}-1\right)\times 100\%$, where the superscripts (+ and −) stand for after and before the addition of Ag^+^ ions (or the addition of LB medium for the controls), respectively. The relative changes for the untreated (orange squares) and Ag^+^-treated bacteria (blue circles) from the full-length trajectories are shown in Figure 7A. Performing a one-sample t-test showed that the increase in $P_{T}$ and decrease in $k_{TR}$ were much more statistically significant for the Ag^+^-treated bacteria (p-values: $2.1\times 10^{-4}$ and $4.1\times 10^{-10}$ for $P_{T}$ and $k_{TR}$, respectively) than the untreated cells (p-values: 0.065 and 0.014, respectively). A two-sample t-test showed that the differences between the treated and untreated samples were also statistically significant (e.g., the p-value for $k_{TR}$ was $4.2\times 10^{-4}$).

Finally, we examined the dependence of the HMM parameters on the treatment time (Figure 7B,C), which was carried out by analyzing individual segments of the full-length ω-trajectories (window-size = 10,000 frames, stride between segments = 5000 frames) using the fitted/trained HMM models. We observed that both $\delta_{P_{T}}$ and $\delta_{k_{TR}}$ started from ≈0, which is reasonable as it takes time for Ag^+^ ions to diffuse to the bacteria and affect the bacteria. More interestingly, the effects of Ag^+^ ions became more and more significant after ∼300 s compared to the controls (Figure 7B,C). After ~750 s, the relative change in $k_{TR}$ reached ∼−90%, suggesting that Ag^+^ ions prevented the flagellar motor of the bacteria from rotating effectively and efficiently.

Comparing the diffusion time scale of Ag^+^ ions and the response time of bacterial rotation to Ag treatment suggests that Ag^+^ ions directly interact with bacterial flagella. As the Ag^+^ ions were added to the top surface of the liquid medium in the Petri dish above the bacteria under observation, the distance that the Ag^+^ ions need to travel to the bacteria is roughly Δx= 0.2 cm (estimated from the volume of the culture medium, 2 mL, and the diameter of the Petri dish, 3.5 cm). Considering that the diffusion coefficient of Ag^+^ ions in water [56] is in the order of $D=1.5\times 10^{-5}$ cm^2^/s, the time scale for the Ag^+^ ions to reach the bacteria is in the order of $\Delta t=\frac{\Delta x^{2}}{6D}\approx$ 400 s, which is close to the response time (300–750 s) of bacteria to the Ag^+^ ions that we measured from our tethering assays (Figure 7B,C). If the observed effects of Ag^+^ ions on the bacterial motility were due to indirect interactions, such as those through regulatory proteins and membrane damages, the response time is expected to be longer as time is needed to transduce those indirect effects to the flagellar motor. Therefore, this simple estimate and comparison suggest that one should focus on the flagellar proteins when searching for the molecular basis of the Ag-caused motility change and motor stalling in future studies.

## 3. Materials and Methods

### 3.1. Bacterial Strain and Growth

An *E. coli* K12-derived strain from Refs. [15,25,39,57] was used in this study. The strain was used in previous investigations of the antimicrobial activities of Ag^+^ ions and AgNPs [15,25,39]. This strain has the *hns* gene knocked out from the chromosomal DNA but supplemented with a plasmid encoding for the H-NS protein fused to the mEos3.2 fluorescent protein [58] and for resistance to kanamycin and chloramphenicol [15,25,39,57].

Each experiment started by inoculating a single bacterial colony into 5 mL of a Luria Broth (LB) medium supplemented with kanamycin and chloramphenicol (50 µg/mL and 34 µg/mL, respectively) [25]. The liquid culture was grown at 37 °C in a shaking incubator (250 RPM) overnight. On the second day, the overnight culture was diluted by 5000× into 5 mL of the fresh LB medium with the antibiotics. The new culture was grown at 32 °C [59,60,61] in the shaking incubator until the bacterial culture reached the mid-exponential phase (OD600 ≈ 0.3), followed by measurements as described below.

### 3.2. Phase-Contrast Microscopy

Measurements in the swimming and tethering assays were conducted at room temperature using phase-contrast microscopy on an Olympus IX-73 inverted microscope equipped with a 100×, NA = 1.25 phase-contrast, oil-immersion objective (Olympus) and an EMCCD camera (Andor Technology). The microscope and data acquisition were controlled using Micro-Manager [62,63]. The effective pixel size of recorded images/movies was 0.16 µm.

### 3.3. Swimming Assay

In swimming assay experiments, *E. coli* bacteria at OD_600_ ≈ 0.3 were treated with Ag^+^ ions at 40 µM for 1, 2, and 4 h, which clearly showed suppressed growth. At each time point, 2 mL of the bacterial culture were transferred to a cleaned glass-bottom Petri dish, followed by monitoring and recording the free swimming of the bacteria using phase-contrast microscopy. The swimming of untreated bacteria (i.e., before the addition of Ag^+^ ions, or 0 h) was monitored and used as a negative control. The exposure time was set to 30 ms, while the actual time interval between adjacent frames of the acquired movies was 54 ms. The acquired movies of freely swimming bacteria were processed in ImageJ by inversion, smoothing, and background subtraction [64,65], followed by automated identification and localization of the bacteria using custom-written MATLAB programs [66]. The localizations of the bacteria were then linked to trajectories following standard algorithms [66,67,68], using a maximum displacement between adjacent frames of 1.92 µm (12 pixels), a memory of 0 frame (i.e., no gap), and a minimum length of 12 frames. The identified trajectories further went through a manual quality-control process by removing the bacteria that were stuck on the glass surface or formed large clumps.

The trajectories of the bacteria in the freely swimming assays were further analyzed using custom-written or open-source Python programs. For example, the instantaneous velocities were calculated from the trajectories r(t) of the bacteria, $v\left(t\right)=\left|\frac{r\left(t+\Delta t\right)-r\left(t\right)}{\Delta t}\right|,$ where Δt=54 ms. In addition, we estimated the maximum chord-to-arc ratio ($\gamma_{CA}^{M}$) for each trajectory, inspired by TumbleScore [35]: $\gamma_{CA}^{M}=\frac{\underset{i,j}{max}\left(\left|r_{i}-r_{j}\right|\right)}{\sum_{i}\left|r_{i+1}-r_{i}\right|},$ where $r_{i}$ and $r_{j}$ are positions of a single trajectory. Furthermore, the changing rates of swimming directions *Ω* were estimated directly from the velocities [35,41,42]: $\Omega_{i}=cos^{-1}\left(\frac{v_{i+1}\cdot v_{i}}{\left|v_{i+1}\right|\left|v_{i}\right|}\right)$. Lastly, we calculated the ensemble mean-square displacement (MSD) for each sample using the *trackpy* Python package [68]: $MSD\left(\tau \right)=\langle {\left(r\left(t+\tau \right)-r\left(t\right)\right)}^{2}\rangle ,$ where τ is the lag time.

### 3.4. Tethering Assay

In the tethering assay experiments [43,44], *E. coli* bacteria in the mid-exponential phase (OD_600_ ≈ 0.3) were tethered to glass-bottom Petri dishes through their flagella. The tethering was achieved by coating the glass surface with biotinylated BSA, neutravidin, and biotinylated anti-FliC antibody sequentially [45,69]. *E. coli* flagella bind to the anti-FliC antibody [70,71], immobilizing the bacteria. The rotations of the tethered bacteria were monitored and recorded under phase-contrast microscopy with an exposure time of 5 ms for 10,000 frames without Ag^+^ ions (the actual time interval between adjacent frames was 14.1 ms). Then, Ag^+^ ions were directly added to the Petri dish at a final concentration of 40 µM, followed by recording the rotations of the same bacteria for 50,000 to 100,000 frames. For negative controls, the LB medium (instead of Ag^+^ ions) was added to the Petri dish and the rotations of the bacteria were recorded similarly. Experiment replications were performed independently on different days.

Bacteria in the tethering assays were identified and characterized in each frame of the recorded movies using custom-written Python programs based on the *scikit-image* package [72]. From the primary axis of the identified bacteria, the orientation θ of the bacteria was obtained [73,74], and then the angular velocities of the bacterial rotation were estimated: ω=Δθ/Δt, where Δt=0.0141 s. We noted that frames containing other non-tethered bacteria invading the region of the tethered ones were removed from further analysis to ensure accuracy. The ω-trajectories were analyzed using the hidden Markov model (HMM) [54], in which two states of the bacteria (running and tumbling/pausing) were assumed. In addition, Gaussian emission distributions were applied for the emission from the two states to the observable values (i.e., angular velocities ω) [54]. HMM analysis was conducted using the *hmmlearn* Python package. For each bacterium in the tethering assay, we fitted the HMM model using the ω-trajectory before the addition of Ag^+^ ions (or the LB medium). Then, the fitted model was used to predict the states for the data after the addition of Ag^+^ ions (or LB medium), from which the probabilities of the two states and the transition rates were estimated [75].

### 3.5. Untethered Rotation Assay

The rotation assay was performed on *E. coli* bacteria with a shortened flagellar by shearing, following published protocols [47,49,50,53,76]. Briefly, *E. coli* bacteria in the mid-exponential phase (OD_600_ ≈ 0.3) were harvested in an ice-cold syringe with a 25-guage needle. The bacteria culture was pushed out to a clean, ice-cold Petri dish and sucked back into the syringe. This process was repeated 50 times, and the resultant bacteria were used for phase-contrast microscopy. Data analysis was performed in the same way as in the tethering assay.

### 3.6. Statistical Analysis

The swimming assay, tethering assay, and untethered rotation assay were repeated multiple times on different days. The number of experiment replications of the assays and the sample sizes (i.e., number of bacteria) are shown in Table 1.

## 4. Conclusions

To conclude, we directly visualized and investigated the antibiotic effects of Ag^+^ ions on the motility of *E. coli* bacteria based on the swimming assay, tethering assay, and untethered rotation assay. Upon exposure to Ag^+^ ions, *E. coli* bacteria slowed down dramatically, showing higher changing rates of swimming directions and increased frequencies of tumbling/pausing of the flagellar motors. Hidden Markov model (HMM) analysis showed that the transition rate from the tumbling/pausing state to the running state decreased, suggesting that Ag^+^ ions stalled the flagellar motors and prevented them from rotating. These experimental observations confirmed our hypothesis that the motility of bacteria is significantly affected by Ag.

This work provides a direct visualization of Ag’s effects on bacterial movements and quantitatively advances our understanding of the mechanism of Ag-based antimicrobial agents in terms of bacterial motility. More importantly, it raises more interesting questions worth further investigations, including the molecular basis for the lower motility caused by Ag and possible bacterial adaptation and resistance against Ag. Addressing these biological questions experimentally is expected to be of great importance and interest for understanding the fundamental antimicrobial mechanism of Ag and further exploring their potential biomedical applications.

## Figures and Tables

**Table 1:** Numbers of replicates of the assays and the sample sizes.

Assay	Replicate	Sample Size	
Swimming Assay	5	0 µM, 0 h: 5906	40 µM, 0 h: 1872
		0 µM, 1 h: 6579	40 µM, 1 h: 2921
		0 µM, 2 h: 588	40 µM, 2 h: 2108
		0 µM, 4 h: 1502	40 µM, 4 h: 2028
Tethering Assay	10	0 µM: 10	40 µM: 15
Untethered Rotation Assay	3	18	

## Data Availability

The data presented in this study are available on request from the corresponding author.

## Supplementary Materials

The following supporting information can be downloaded at: https://www.mdpi.com/article/10.3390/ijms241411704/s1.
